# High-Voltage Insulation Organic-Inorganic Nanocomposites by Plasma Polymerization

**DOI:** 10.3390/ma7010563

**Published:** 2014-01-20

**Authors:** Wei Yan, Zhao Jun Han, B. Toan Phung, Franz Faupel, Kostya (Ken) Ostrikov

**Affiliations:** 1 School of Electrical Engineering and Telecommunications, The University of New South Wales, Kensington, NSW 2052, Australia; E-Mails: winston.wei.yan@outlook.com (W.Y.); toan.phung@unsw.edu.au (T.P.); 2 CSIRO Materials Science and Engineering, Lindfield, NSW 2070, Australia; E-Mail: zhaojun.han@csiro.au; 3 Institute for Materials Science, Synthesis and Real Structure, Faculty of Engineering, Christian-Albrechts-University (CAU) Kiel, Kiel 24118, Germany; E-Mail: ff@tf.uni-kiel.de; 4 School of Physics, The University of Sydney, Sydney, NSW 2006, Australia

**Keywords:** organic-inorganic nanocomposites, plasma polymerization, electrical insulation, dielectric constant

## Abstract

In organic-inorganic nanocomposites, interfacial regions are primarily influenced by the dispersion uniformity of nanoparticles and the strength of interfacial bonds between the nanoparticles and the polymer matrix. The insulating performance of organic-inorganic dielectric nanocomposites is highly influenced by the characteristics of interfacial regions. In this study, we prepare polyethylene oxide (PEO)-like functional layers on silica nanoparticles through plasma polymerization. Epoxy resin/silica nanocomposites are subsequently synthesized with these plasma-polymerized nanoparticles. It is found that plasma at a low power (*i.e.*, 10 W) can significantly increase the concentration of C–O bonds on the surface of silica nanoparticles. This plasma polymerized thin layer can not only improve the dispersion uniformity by increasing the hydrophilicity of the nanoparticles, but also provide anchoring sites to enable the formation of covalent bonds between the organic and inorganic phases. Furthermore, electrical tests reveal improved electrical treeing resistance and decreased dielectric constant of the synthesized nanocomposites, while the dielectric loss of the nanocomposites remains unchanged as compared to the pure epoxy resin.

## 1. Introduction

The application of organic-inorganic nanocomposites has grown rapidly in various fields, such as drug delivery, bio-degradable food packaging, high density energy storage, thermal resistive materials, *etc*. [1–6]. Recently, it has been reported that nanocomposites also possess superior dielectric properties, which are of significant interest to the electrical engineering community [5,7,8]. In general, the dielectric properties are not only related to the characteristics of the constituent components of the nanocomposite, but also to the interactions at the interfacial region between the nanoparticles and the host polymers [9]. However, a common problem associated with organic-inorganic nanocomposite systems is the incompatibility between the two vastly distinct phases, which causes inhomogeneous dispersion of the nanofillers and poor filler-matrix interactions, leading to mediocre insulation performance.

In order to maximize the performance of nanocomposites, surface properties of the nanoparticles need to be carefully tailored before mixing them together. It has been widely reported that the use of wet-chemical methods can graft reactive organic groups onto inorganic nanoparticles, leading to improved affinity with the surrounding polymer matrices [10]. However, large-scale application of wet-chemical processes is limited by their complexity, long-time procedure, low-energy efficiency, and environmental hazards.

In light of the demand of more efficient and environment-friendly approaches, we propose to functionalize the surface of nanoparticles via plasma polymerization. This approach is motivated by the versatility and feasibility of plasma in modifying surfaces of nanomaterials [11], as well as the proven fact that polymeric nanocomposites synthesized with plasma polymerized nanoparticles possess superior physical and chemical properties [12,13]. In this study, dielectric nanocomposites are fabricated with hydrophilic epoxy resin and SiO_2_ nanoparticles. Prior to being introduced to the host polymers, the SiO_2_ nanoparticles are modified by plasma polymerization with diglyme monomers. After the treatment, it is shown that the surfaces of nanoparticles can be coated with polyethylene oxide (PEO)-like layers, which may give rise to the hydrophilicity of the nanoparticles and enable the formation of C–O covalent bonds with the polymer molecules. Electrical insulation tests of such nanocomposites demonstrate the enhanced resistance against electrical treeing, which is a typical fault in high voltage power system equipment. These behaviors can significantly benefit the application of this kind of nanocomposites on electrical insulation. Moreover, we also discuss the important roles of plasma polymerization in modifying organic-inorganic nanocomposites.

## 2. Experimental

### 2.1. Materials

Silica nanoparticles (AEROSIL^®^ 380) with a specific surface area of 380 ± 30 m^2^/g were adopted as the nanofillers for synthesizing the nanocomposites. Epoxy resin was used as the host polymer of the nanocomposites. The two-part thermoset epoxy resin consisted of bisphenol-A diglycidyl ether (BADGE) as the pre-polymer resin and triethylenetetramine (TETA) as the curing agent. Moreover, bis(2-methoxyethyl) ether (diglyme) (Sigma Aldrich, Sydney, Australia) was employed as the monomer for plasma polymerization.

### 2.2. Plasma Polymerization

Plasma polymerization was carried out using the electrode system described elsewhere [14]. Specifically, the power was generated by a 350 kHz RF supply with a built-in matching network. The output voltage was elevated by an RF step-up transformer up to 5 kV peak-to-peak. The voltage was fed to the high-voltage electrode suspended in the reaction chamber through a current limiting resistor. The wall of the reactor was grounded. The monomer vapour was then carried by helium via a bubbler at the flowing rate of 100 standard cubic centimetres per minute (sccm). The pressure of the reactor was kept at 1.4 Torr. A cylindrical stainless steel tube with the outer diameter of 6.35 mm was used as the high-voltage electrode, whereas the wall of the vacuum chamber worked as the ground electrode. Such electrode configuration enabled the generation of uniform plasma in the reaction zone. During the treatment, the nanoparticles were loaded in a quartz vial placed at the bottom of the chamber. The high-voltage electrode entered the vial from the top and the distance from the nozzle to the bottom of the vial was kept at 30 mm. To ensure uniform exposure of the nanoparticles to the plasma, magnetic stirring was performed throughout the treatment.

### 2.3. Nanocomposite Preparation

The nanocomposite samples were prepared by dispensing 420 mg plasma-polymerized nanoparticles into 10 g pre-polymer resin. The mixture was then ultrasonicated for 30 min, followed by blending for 30 min. After that, 4 g curing agent was added into the mixture. Then, the slurry was blended for another 30 min. Vacuum degassing was performed on the viscous mixture to eliminate trapped air bubbles. Afterwards, the nanocomposite liquid was dispensed into the pre-made moulds and further degassed. The samples were then cured under room temperature for 48 h. Nanocomposites with as-received SiO_2_ nanoparticles and the pure epoxy resin samples were also prepared as controls.

### 2.4. Materials Characterization

Optical emission spectrometer (OES, Princeton Instruments SP2500, Trenton, NJ, USA) was used to characterize the plasma. The scanned wavelength ranges from 200 to 1100 nm. X-ray photoelectron spectroscopy (XPS, SAGE 150 SPECS, Berlin, Germany) was utilised to analyse the surface chemistry of the plasma polymer layers coated on the nanoparticles as well as the nanocomposites. Moreover, scanning electron microscopy (SEM, Zeiss Auriga, Jena, Germany) was used to investigate the dispersion property and transmission electron microscopy (TEM, JOEL 2100, Tokyo, Japan) was used to verify the plasma polymer layers coated on the nanoparticles. A thin layer of polycrystalline Au nanoparticles were sputtered on Cu grids prior to TEM examination. Plasma polymers were then coated on the grid under the same condition as for the SiO_2_ nanoparticles. The experiment was designed in this way because of the amorphous nature of silica nanoparticles, which makes it indistinguishable with the amorphous polymer coating under TEM.

Electrical treeing tests of the nanocomposites were performed to reveal the electrical insulation property of the nanocomposites. A pair of needle-to-plane electrodes was employed. An alternating current of 10 kV and 200 Hz was applied on the needle electrode while the plane electrode was grounded. The electrical treeing phenomenon was imaged by a digital camera at the ocular of an optical microscopy with an interval of 10 min. On the other hand, the dielectric properties were investigated by a wide band impedance analyser. The dielectric constant was measured in the frequency range from 20 Hz to 2 MHz. The dielectric loss was obtained in the frequency range from 50 kHz to 2 MHz.

## 3. Results and Discussion

### 3.1. Characterization of Plasma Polymers

**Figure 1.**
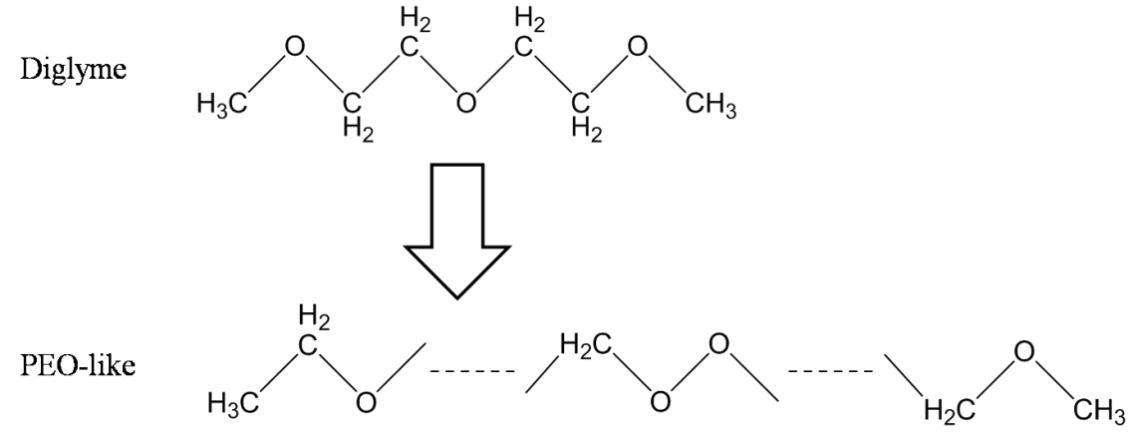
The chemical reaction of the plasma polymerization of polyethylene oxide (PEO)-like polymer.

The plasma polymerization process of the diglyme monomer is demonstrated in Figure 1. Specifically, energetic electrons in the RF plasma could break the C–O and C–C bonds of the diglyme molecules. Then, recombination of the fragments took place on the substrate and formed cross-linked polymer layers with non-repetitive units. The chain of the plasma polymer mainly consisted of –C–C–O–, –C–O–O–, *etc.*, which was similar to the backbone structure of poly-ethylene oxide (PEO), a widely used polymer for non-fouling biocompatible surfaces [15]. Therefore, we denote this thin plasma polymer layer as PEO-like polymer.

**Figure 2.**
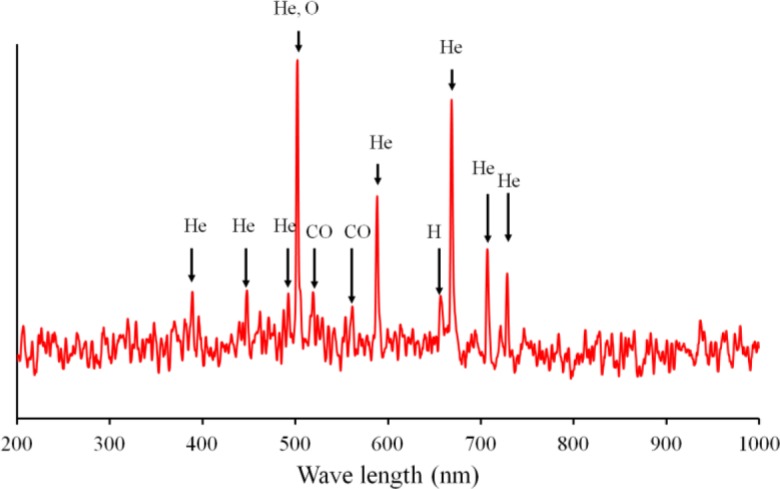
Optical emission spectrometer (OES) of the plasma for synthesizing the PEO-like polymer.

**Figure 3.**
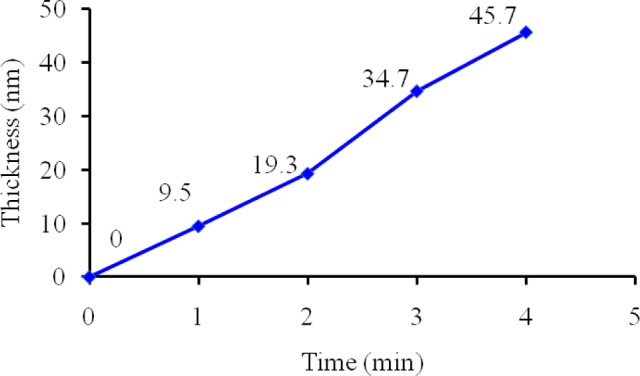
Thickness of the PEO-like film as a function of the plasma polymerization time.

**Figure 4.**
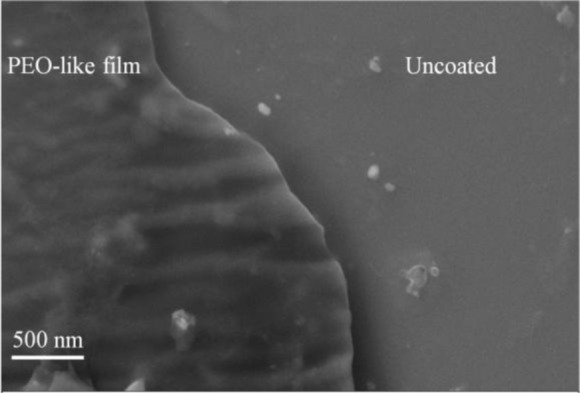
Surface morphology of the plasma polymerized PEO-like film.

To investigate the plasma polymerization process, the OES spectrum of helium/diglyme plasma was obtained (Figure 2). Peaks corresponding to He (389, 447, 492, 588, 668, 707, and 728 nm), O (502nm), H (656nm), and CO radicals (519 and 561 nm) can be clearly identified. In particular, the presence of CO radical may render the possibility of synthesizing PEO-like polymers [16]. On the other hand, the growth rate of the PEO-like film was about 11.5 nm/min under the given experimental condition (Figure 3). The surface morphology of the plasma polymerized film was studied using SEM. The edge between the PEO-like film and the masked part of the substrate, which was a glass slide, is clearly shown in Figure 4. One can see that uniform coating can be achieved through plasma polymerization. Such characteristic is highly favourable in modification of nanoparticles, where homogeneous shell layers on each of the nanoparticles are desired.

**Figure 5.**
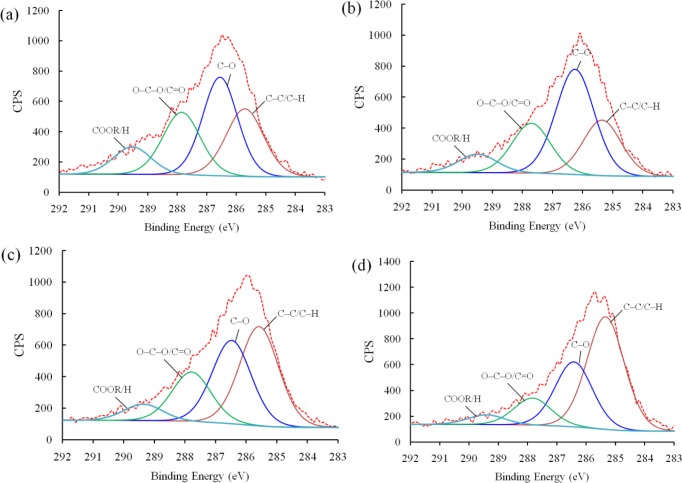
XPS spectra of *C 1s* peaks of plasma polymerized PEO-like film at (**a**) 5 W; (**b**) 10 W; (**c**) 15 W; and (**d**) 20W for 4 min.

Next, XPS analysis was carried out to investigate the chemical bonding of the plasma polymerized film. Figure 5 shows the deconvoluted *C 1s* peaks of PEO-like films synthesized for 4 min at different powers under which the plasma was generated, *i.e.*, 5, 10, 15, and 20 W, respectively. The O/C ratios of the PEO-films were 0.27 (5 W), 0.31 (10 W), 0.2 (15 W), and 0.23 (20 W). Therefore, the polymer synthesized under a moderate power (10 W) possessed fairly good retention of property, of which the O/C ratio is 0.5 [17]. With the XPS narrow scans, four peaks can be deconvoluted from the *C 1s* band of each sample. The binding energies, corresponding chemical bonds, and percentage of concentration are given in Table 1. It should be noted that the peak at 286.5 eV corresponding to the ether bonds, which is considered as the characteristic peak of conventional PEO polymers [18], was the highest (45.97%) at 10 W among these four conditions. The carboxyl content resulted from the polar sites led to the increase of hydrophilicity, whereas the hydrocarbon content formed into non-polar groups, which can increase the hydrophobicity. Given the hydrophilic nature of the epoxy resin matrix used in synthesizing the nanocomposites, 10 W was selected as the standard plasma power.

### 3.2. Characterization of Plasma Polymerized Nanoparticles

**Figure 6.**
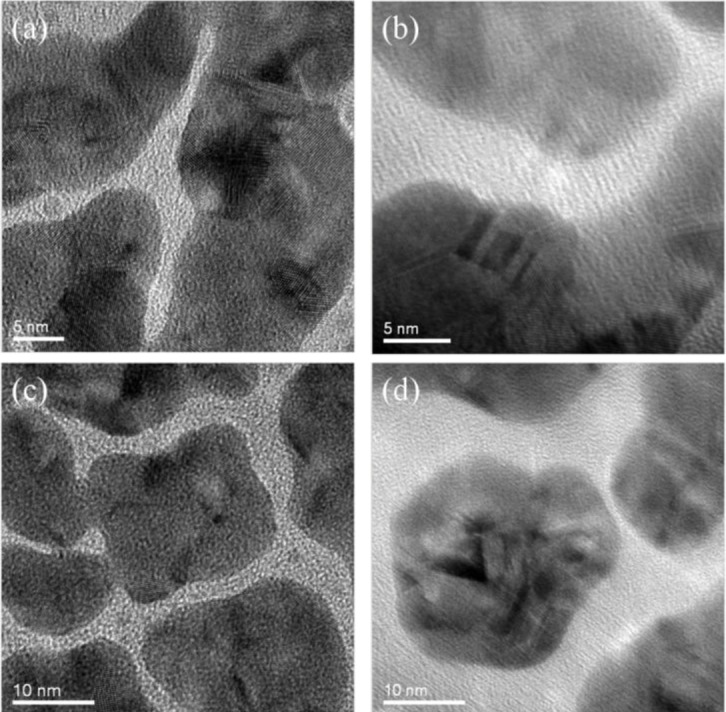
TEM images of plasma polymerized Au nanoparticles. (**a**) and (**c**) are uncoated Au nanoparticles with clear crystalline edges in different resolutions; (**b**) and (**d**) are PEO-like polymer coated Au nanoparticles with amorphous edges in different resolutions.

The surface morphology of nanoparticles coated with the plasma polymerized PEO-like films was characterized using TEM. As mentioned above, this characterization cannot be performed directly on SiO_2_ nanoparticles as both the polymer coating and the SiO_2_ nanoparticle substrate were amorphous, which was difficult to be distinguished in the TEM image. In this case, Au nanoparticles sputtered on Cu grids were adopted as the substrate instead. Figure 6 shows the uncoated and PEO-like film coated Au nanoparticles at different resolutions. One can observe that the crystalline structure of Au nanoparticles exists in all of the areas of the uncoated samples (Figure 6a,c). Also, the edge between the Au nanoparticles and the background (amorphous carbon film) can be readily identified. For the PEO-like film coated ones, however, amorphous layers around the crystalline Au nanoparticles blurred the edge of the nanoparticles (Figure 6b,d). Therefore, the PEO-like film was successfully coated on Au nanoparticles by plasma polymerization. This indicates that similar PEO-like layers were highly likely to be coated on SiO_2_ nanoparticles.

### 3.3. Characterization of Nanocomposites

**Figure 7.**
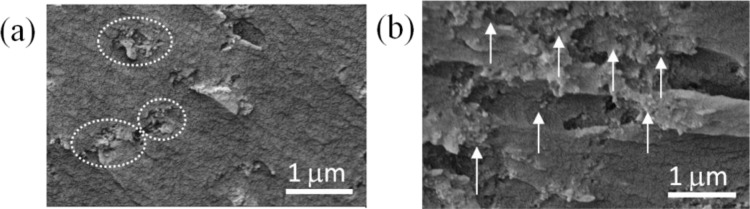
SEM images of (**a**) the nanocomposite with as-received nanoparticles; and (**b**) the nanocomposite with plasma polymer coated nanoparticles. Nanoparticle agglomerations are marked in (**a**) by dotted circles and well-dispersed nanoparticles are pointed by arrows in (**b**).

The dispersion uniformity of the nanoparticles in the epoxy/SiO_2_ nanocomposites was analysed with the SEM images on the cross-section of the nanocomposites, as shown in Figure 7. One may find that the nanoparticles formed into clusters with diameters ranging from a few hundred nanometres to 1 micrometre in the nanocomposites blended with as-received SiO_2_ nanoparticles (Figure 7a). In the nanocomposites with plasma polymer coated nanoparticles, however, the dispersion was improved as the average inter-particle distance becomes more and no obvious nanoparticle agglomerations or clusters were observed (Figure 7b). The improvement of the dispersion can be attributed to the high O/C ratio (0.31) and high concentration of C–O groups (45.9%) as described above, which rendered more hydrophilic surfaces on the SiO_2_ nanoparticles.

**Figure 8.**
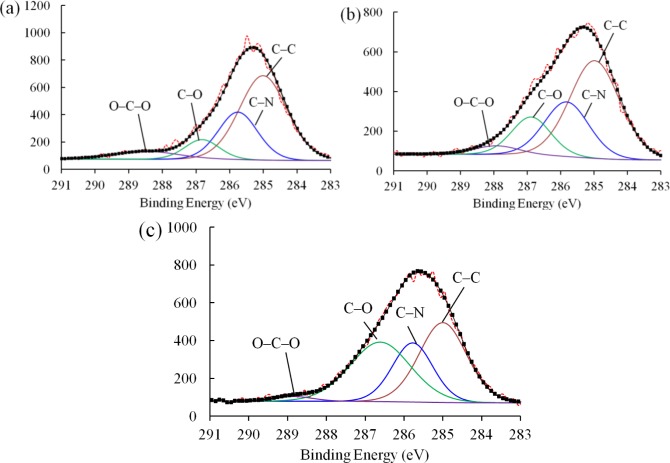
*C 1s* peaks of the deconvoluted XPS spectra of (**a**) the pure epoxy resin; (**b**) the nanocomposite with as-received nanoparticles; and (**c**) the nanocomposite with plasma polymer coated nanoparticles.

Figure 8 shows the *C 1s* peaks of the XPS spectra for the pure epoxy resin, nanocomposites with as-received SiO_2_ nanoparticles, and nanocomposites with plasma polymer coated SiO_2_ nanoparticles. For each material, the spectrum can be deconvoluted into four characteristic peaks. Specifically, the peak of the lowest binding energy was assigned to the C–C/C–H bonds. Due to the insulating nature of the samples, positive shifts presented on each of the spectra [19]. We therefore calibrated the spectrum by fixing the C–H/C–C peak to 285 eV. Additionally, the peaks at around 285.7, 286.8, and 288 eV can be attributed to the C–N, C–O, and C–O–C bonds, respectively. The exact values of the binding energies of each material and the concentrations of corresponding bonds are given in Table 2.

**Figure. 9.**
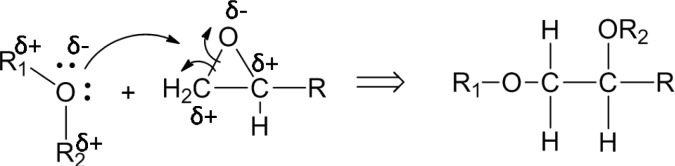
Chemical reaction between oxygen-containing surface groups and the epoxide ring or the pre-polymer epoxy resin.

The concentrations of each bond in the nanocomposites with as-received SiO_2_ nanoparticles remained similar to that of the pure epoxy resin, indicating that the inclusion of the as-received SiO_2_ nanoparticles failed to improve the interfacial structure of the nanocomposite due to the poor filler-matrix interaction. However, the concentration of C–O bonds of the nanocomposites with plasma polymer coated SiO_2_ nanoparticles (36.15%) increases dramatically as compared to that with as-received nanoparticles (16.14%). This can be explained by the nucleophilic substitution reaction between the surface oxygen-containing functional groups on the nanoparticles and the epoxide rings in the pre-polymer resin. Figure 9 shows the proposed process of reaction. The two lone pairs of oxygen atoms may act as nucleophilic sites, which can readily react with the electrophilic carbon atoms in the epoxide ring. Although nucleophilic substitution reactions normally take place in acidic environments, the epoxide ring can be cleaved without acidic catalysts due to the high strain in the three-member ring. As a result, the carbon atoms at the end of the epoxide ring may form C–O bonds with the oxygen atoms at the surface group of the nanoparticles. In this way, the polymer molecules can be anchored to the surface of the nanoparticles. Consequently, the number of C–O functional groups in the nanocomposite was increased significantly.

The interaction between nanoparticles and epoxy resin can also be affected by the hydrogen bonds formed between the two phases. As the plasma polymerization process imparted oxygen-containing groups, which were electronegative onto the surface of the nanoparticles, more hydrogen atoms from the molecule of the epoxy resin can be attracted to the nanoparticles through hydrogen bonds. It was possible that these two types of reaction happened during the mixing of the nanoparticles and the pre-polymer resin. Each of the nanoparticles was “dragged” away by adjacent epoxy molecules through the above mechanisms. As the resin is fluidic, the dispersion of nanoparticles can be further improved by stirring.

**Figure 10.**
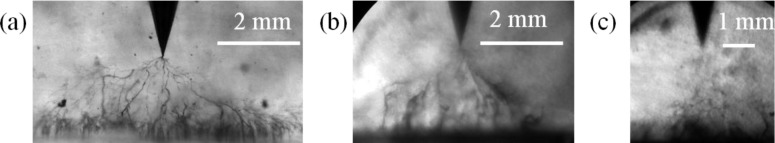
Electrical trees before breakdown of (**a**) the pure epoxy resin, (**b**) the nanocomposite with as-received nanoparticles, and (**c**) the nanocomposite with plasma polymer coated nanoparticles.

The electrical treeing testes of these nanocomposites are shown in Figure 10. It was found that the tree initiated in the pure epoxy resin sample in 10 min after the application of a high voltage. The ultimate breakdown occurred at 89 min (Figure 10a). The radial extent of the tree, which describes the diameter of the fully developed tree before the sample is broken down [20], was around 7 mm.

Similarly, the electrical tree was initiated in 10 min in the nanocomposites with as-received silica nanoparticles. In general, the electrical trees in organic-inorganic nanocomposites should have a higher bush-like character as compared to the pristine polymers [21]. However, due to the poor dispersion of nanoparticles, thick branches are observed in the present case with as-received silica nanoparticles (Figure 10b). This sample failed after 79 min and the radial extent of the tree was approximately 4 mm.

In contrast, the electrical tree for the nanocomposite with plasma polymer coated silica nanoparticles did not initiate in the first 50 min. After that, the tree continued growing and more sub-branches were formed with high branch density. The ultimate breakdown of this sample took place at 100 min, which was the longest among all samples. The radial extent was around 2 mm, which was also the smallest value (Figure 10c).

**Figure 11.**
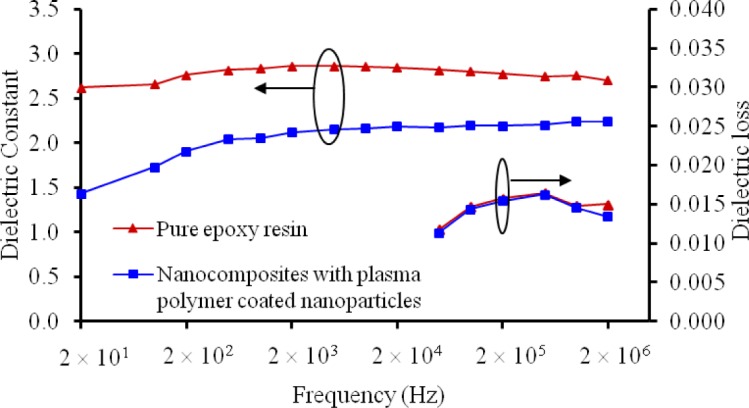
Comparison on dielectric constant and dielectric loss of the pure epoxy resin, nanocomposite with as-received silica nanoparticles, and nanocomposites with plasma polymer coated silica nanoparticles.

The dielectric constant *k* and the dielectric loss (tanδ) of the nanocomposites with plasma polymerized nanoparticles were also investigated, as plotted in Figure 11. One can see that the *k* value of the nanocomposite with plasma polymerized silica nanoparticles was dramatically lower than the pure epoxy resin. The reduced *k* values of the nanocomposites can be attributed to the restriction on the molecular mobility [22]. According to the parallel model of dielectrics, the dielectric loss can be expressed by Equation (1).

$$\text{tan}\delta =1/(\omega R_{\text{p}}C_{\text{p}})$$ (1)

where *R*_p_ and *C*_p_ are the parallel resistance and capacitance of the dielectric, respectively. As the dielectric constant is proportional to *C*_p_, the decrease of *k* should be accompanied by a higher tanδ. In our results, however, the two types of materials had a similar dielectric loss at all frequencies. The exact mechanism is unclear. Nevertheless, we speculate that the bonds created between the nanoparticles and the polymer matrix can restrain dipole movements. This may thus increase the parallel resistance *R*_p_ and offset the reduction in *C*_p_. As such, the dielectric loss of the nanocomposites with the plasma polymerized nanoparticles was maintained at a similar level as that of the pure epoxy resin.

## 4. Conclusion

Although plasmas have been widely used in surface modification of nanomaterials, little work has been reported on their application in fabricating high-performance dielectric nanocomposites. In this paper, we have explored a novel method which uses plasma polymerization technique to coat PEO-like layers on silica nanoparticles. It has been found that the surface chemistry of plasma polymer layers was largely influenced by the plasma power. Low-power density of the plasma zone may cause insufficient dissociation of the monomers, whereas high power may result in excessive molecule fragmentation. In this case, nanoparticles treated with a medium plasma power (10 W) were adopted for synthesizing nanocomposite dielectrics. It has been found that the dispersion uniformity improved as the hydrophilicity of the silica nanoparticle increased. The plasma polymerized surface also provided rich anchoring sites for the polymer molecules, enabling the formation of C–O bonds in the interfacial regions. These changes in the chemistry of the nanocomposites have resulted in significant improvements in their dielectric properties, in particular, the reinforced resistance against electrical treeing. These results demonstrated the capability of plasma polymerization in modifying organic-inorganic nanocomposites for advanced applications in electrical insulation.

## Figures and Tables

**Table 1.** *C 1s* binding energy and surface concentration from the XPS spectra of the plasma polymerized PEO-like film at 5 W, 10 W, 15 W, and 20 W.

Binding Energy (eV)	Chemical Bonds	Concentration%			
		5 W	10 W	15 W	20 W
289.2	COOR/H	11.08	8.07	6.66	4.51
288	O–C–O/C=O	25.16	21.78	19.91	12.62
286.5	C–O	36.67	45.97	33.46	30.36
285	C–C/C–H	27.08	24.19	39.96	52.52

**Table 2.** Characteristics of the peaks in *C 1s* spectra for pure epoxy resin, nanocomposite with as-received nanoparticles, and nanocomposite with plasma polymer coated nanoparticles.

Sample	Binding Energy (eV)	Concentration (%)	Groups
Pure epoxy resin	285.00	56.86	C–C/C–H
	285.76	26.47	C–N
	286.82	9.49	C–O
	288.49	7.18	C–O–C

Nanocomposite with as-received silica	285.00	53.08	C–C/C–H
	285.83	26.67	C–N
	286.89	16.14	C–O
	287.93	4.11	C–O–C

Nanocomposite with plasma polymer coated silica	285.00	37.96	C–C/C–H
	285.78	24.02	C–N
	286.61	36.15	C–O
	288.84	1.87	C–O–C
